# Debulking surgery for venous hemangioma arising from the epicardium: report of a case

**DOI:** 10.1186/s12957-017-1152-1

**Published:** 2017-04-12

**Authors:** Daichi Shikata, Takahiro Nakagomi, Yujiro Yokoyama, Yukiko Yamada, Masato Nakajima, Toshio Oyama, Taichiro Goto

**Affiliations:** 1 Department of General Thoracic Surgery, Yamanashi Central Hospital, Yamanashi, Japan; 2 Department of Cardio-Thoracic Surgery, Yamanashi Central Hospital, Yamanashi, Japan; 3 Department of Pathology, Yamanashi Central Hospital, Yamanashi, Japan; 4 Lung Cancer and Respiratory Disease Center, Yamanashi Central Hospital, Yamanashi, 400-8506 Japan

**Keywords:** Cardiac hemangioma, Epicardium, Diagnosis, Surgery

## Abstract

**Background:**

Cardiac hemangiomas are rare benign vascular tumors that can occur in any cardiac layer: endocardium, myocardium, or epicardium. Histologically, cardiac hemangiomas may be classified as capillary, cavernous, or arteriovenous; venous hemangiomas are extremely rare.

**Case presentation:**

A 46-year-old man reported experiencing precordial discomfort. Computed tomography revealed a massive tumor adjacent to the right ventricle. The right coronary artery was found to be located at the center of the tumor. Cardiovascular angiography showed that the artery was completely encased by the tumor without any obstruction and that the right ventricular lumen was compressed by the tumor. Surgical debulking of the tumor was performed under cardiopulmonary bypass, and the frozen section led to a diagnosis of benign hemangioma. The tumor was debulked as much as possible until the right coronary artery appeared. For decompression of the heart, the pericardium was left open to the thoracic cavity and unsutured. Histopathologic examination revealed a diagnosis of epicardial venous hemangioma.

**Conclusions:**

Cardiac hemangioma should be included in the differential diagnosis of mediastinal tumor in reference to the location and flow of the coronary artery. Surgical resection, or at least tumor debulking, is required to confirm the diagnosis and prevent further complications and has a favorable clinical outcome.

## Background

Cardiac hemangiomas are extremely rare, accounting for 1 to 2% of primary benign cardiac tumors, and may arise from any cardiac layer: endocardium, myocardium, or epicardium [1–3]. Most cardiac hemangiomas are asymptomatic and found incidentally; symptoms depend on tumor size and location [4]. Diagnosis can be made by echocardiography, computed tomography (CT), or coronary angiography; however, in most cases, diagnosis is made only after surgical excisional biopsy.

Cardiac hemangiomas are histologically classified into three morphologic subtypes: capillary, cavernous, and arteriovenous. Herein, we report a case of venous cardiac hemangioma, an extremely rare subtype of the disease.

## Case presentation

**Fig. 1:**
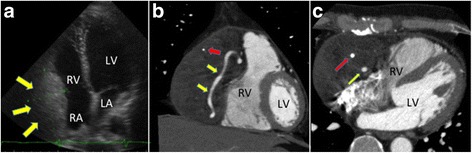
Diagnostic images. **a** Echocardiogram, demonstrating a hyperechoic mass (*yellow arrows*) adjacent to the RV. **b**, **c** Coronal (**b**) and transverse (**c**) coronary computed tomography scans, demonstrating a mass compressing the RV. The *red* and *yellow arrows* indicate calcification and the coronary artery, respectively. Abbreviations: *LA* left atrium, *LV* left ventricle, *RA* right atrium, *RV* right ventricle

**Fig. 2:**
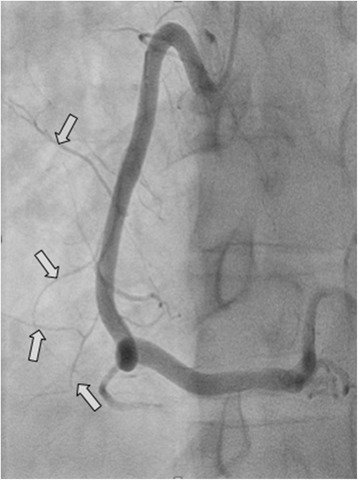
Coronary angiogram, showing the collateral vessels (*arrows*) supplying the tumor from the right coronary artery without any obstruction

A 46-year-old man reported experiencing precordial discomfort and was referred to our department. He was a nonsmoker and had no history of any significant illness or surgery. Physical examination and electrocardiography showed no abnormal findings. Chest radiography, however, showed an enlarged right ventricular shadow, leading to a slightly elevated cardiothoracic ratio of 56%. Transthoracic echocardiography showed a mass in the pericardium, 8.0 × 4.0 cm in diameter, that compressed the wall of the right atrium and ventricle (Fig. 1a); left ventricular function was within normal limits. There was no tricuspid regurgitation or stenosis. Contrast-enhanced chest CT showed a low-density mass in the pericardium that compressed the wall of the right atrium and ventricle and encased the right coronary artery (Fig. 1b, c). The mass was relatively homogenous except for calcified nodules (Fig. 1c). No clear separation could be seen between the ventricles and the mass. Coronary angiography showed that the mass had some feeding arteries and that the proximal and mid-portions of the right coronary artery were inside of the tumor without any obstruction (Fig. 2). No other signs of coronary disease were observed. Laboratory test results, including biochemistry, coagulation, routine hematology, and serum tumor markers, were all within normal limits.

The differential diagnosis included sarcoma or primary lymphoma, though the epicardial location would have been atypical for both tumors. Subsequently, biopsy and surgical resection of the tumor were scheduled in order to establish a precise diagnosis and to improve the outflow from the right ventricle and, thus, his symptomatic condition.

Median sternotomy was completed under general anesthesia. During surgery, the tumor was determined to be hemorrhagic because incisional biopsy alone caused active bleeding, and cardiopulmonary bypass was initiated. Frozen section led to a diagnosis of a benign vascular tumor, but the right coronary artery was totally encased by the tumor, which was judged to be not amenable to curative resection. Thus, the tumor was resected directly with an electrical scalpel and debulked to the proximity of the coronary artery. The resected tumor specimen appeared as a spindle with a maximum diameter of 6 cm, accounting for approximately 50% of the original tumor volume.

**Fig. 3:**
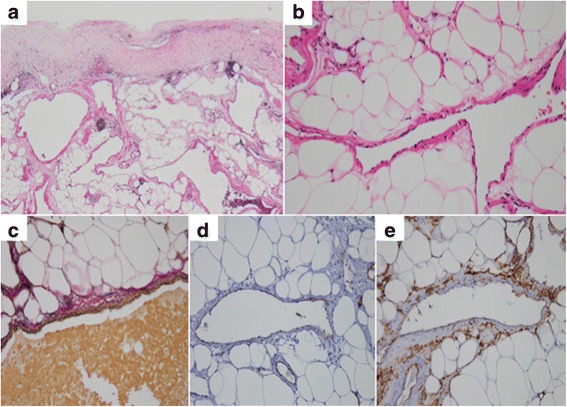
**a**–**c** Histopathologic findings, showing characteristic venous structures widely in the fat layer of the pericardium. A vascular lumen is formed by a vein containing thin elastic fibers (**a**, **b** hematoxylin and eosin stain; **c** Elastica van Gieson stain). **d**, **e** Immunohistochemical staining with endothelial markers CD31 (**d**) and CD34 (**e**), confirming the presence of a thin internal layer of endothelial cells

Histopathologically, dilated venous vessels with smooth muscle layer were widely observed in the fat layer of the pericardium (Fig. 3a–c), leading to a diagnosis of venous cardiac hemangioma. On immunohistochemical staining, endothelial markers CD31 and CD34 were positive (Fig. 3d, e).

**Fig. 4:**
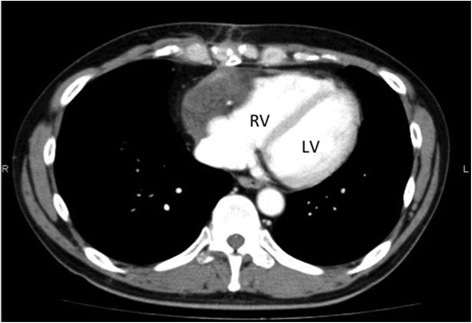
Postoperative chest computed tomography scan, showing volume reduction of the tumor and improvement of the compression of the RV. Abbreviations: *LV* left ventricle, *RV* right ventricle

The patient had an uneventful postoperative course and was discharged on postoperative day 7. Follow-up chest CT showed volume reduction of the tumor and improvement of the compression of the right ventricular wall, and no subsequent regrowth (Fig. 4). Presently, 36 months after surgery, the patient is living a healthy life without any symptoms.

## Discussion

Primary cardiac tumors are rare, with a reported incidence of 0.0017 to 0.27% at autopsy. Hemangiomas are thought to represent only 1 to 2% of benign cardiac tumors [1–3]. Hemangiomas can occur at any age, and there is no sex predominance. Hemangiomas may arise from any cardiac layer—endocardium, myocardium, or epicardium—and are commonly located on the left or right ventricular free wall or septum [1, 5].

Cardiac hemangiomas are generally classified into three histologic subtypes: capillary (small vessels resembling capillaries), cavernous (multiple dilated thin-walled vessels), and arteriovenous (dysplastic malformation of arteries and veins). Often, some combination of these subtypes is observed in a tumor. In our case, histopathologic examination confirmed a venous cardiac hemangioma demonstrating small venous vessels in the fat layer of the pericardium. This subtype is not included in the three conventional subtypes and is an extremely rare disease entity.

Most cardiac hemangiomas are asymptomatic and found incidentally, though they may present with dyspnea, palpitation, arrhythmia, murmur, pericardial effusion, or thromboembolic events; symptoms usually depend on tumor size and location [4]. In our case, the patient’s chief complaint was chest discomfort, which was assumed to be attributed to compression of the right ventricle by the tumor because the discomfort resolved spontaneously after surgery. For diagnosis of cardiac hemangioma, echocardiography, CT, and coronary angiography are generally useful. Hemangiomas appear on echocardiography as hyperechoic lesions [6, 7]. Chest CT shows a heterogeneous mass on unenhanced sequence, which is intensely enhanced after contrast administration [8]. In our case, contrast-enhanced chest CT showed the tumor as a low-density mass, which may be explained by the unique histologic subtype of venous hemangioma. The tumor also had calcification, which is thought to have arisen from a thrombus in a dilated vessel. Coronary angiography can be useful in clarifying the distribution and delineating the feeding vessels to the tumor based on the characteristic tumor blush [3, 9]. In our case, coronary angiography showed the feeding arteries from the right coronary artery; the coronary artery was encased by the tumor but was intact without any evidence of stenosis. Thus, the hemangioma in our case was suggested to be a soft tumor.

In many cases, definitive diagnosis of cardiac hemangioma cannot be made preoperatively, as in our case. For cardiac hemangiomas, complete resection is usually the treatment of choice [10–13]. After complete resection, the prognosis is generally favorable with a low rate of recurrence [3, 11, 13, 14]. Even incomplete resection and only tumor debulking, as in our case, is reported to produce long-term survival benefits [1]. We utilized cardiopulmonary bypass because the tumor bled profusely at the slightest biopsy. As observed on preoperative angiography, the tumor extended to encase the coronary artery so that no exact borderline could be detected between the tumor and the normal tissue of the pericardium. Thus, we judged that the tumor was not amenable to curative resection, and we chose tumor debulking. Botha et al. reported a case of cardiac hemangioma involving both ventricles that was judged to be unresectable because of the extent of coronary involvement [15]. In our case, because the right coronary artery ran near the heart side of the tumor, we considered that the part of the hemangioma located lateral from the coronary artery could be resected carefully.

Sclerotherapy is frequently performed as first-line therapy [16]. However, it appears to be inappropriate for large lesions and can produce inflammatory fibrosis and a permanent scar when the chemical agent is directly applied to infiltrated muscles. Moreover, it requires multiple courses and carries a risk of serious complications, such as a pulmonary embolism [17]. We did not perform sclerotherapy during surgery owing to the reasons stated above, but it can be considered as a treatment option if regrowth of hemangioma occurs in the future.

The impressive effect of propranolol in treating infantile hemangiomas has provoked a paradigm shift in their management over the past few years [18, 19]. In 2008, propranolol, a nonselective β-adrenergic antagonist, was serendipitously discovered to cause regression of proliferating hemangiomas in newborns receiving treatment for cardiovascular disease [19]. Numerous studies demonstrating the success of propranolol in shrinking hemangiomas have also been reported thereafter [19–21]. In addition, good tolerance of propranolol has been established [22]. Thus, treatment with propranolol has become the mainstay of systemic therapy for hemangiomas.

Treatment for hemangiomas depends on their size, location, and severity. Given the fact that cardiac hemangiomas are benign, it can be expected that our patient will remain asymptomatic for a long time. Thus, therapeutic strategies and surgical procedures for benign cardiac tumors should be considered flexibly with consideration of the positional relationship of the tumor with the coronary artery, the extent of coronary involvement, the rate of tumor growth, and the age or request of the patient. Moreover, in our case, surgery was completed with the pericardium left open. For benign cardiac tumors, it seems that such an innovative technique should be applied to prevent symptoms of heart compression in case of tumor regrowth. Finally, regular screening with various imaging modalities is highly recommended in all cases, especially for those with incomplete tumor resection, such as our case.

## Conclusions

Cardiac hemangioma is a rare disease entity; however, it should be included in the differential diagnosis of mediastinal tumor in reference to the location and flow of the coronary artery. Surgical resection, or at least tumor debulking, is required to confirm the diagnosis and prevent further complications and has a favorable clinical outcome.

## Abbreviation

CT: Computed tomography
